# Molsidomine ameliorates diabetic peripheral neuropathy complications in Wistar rats

**DOI:** 10.1002/ame2.12162

**Published:** 2021-03-23

**Authors:** Pranav Nayak B, Nathani Minaz, Khadar Pasha

**Affiliations:** ^1^ Pharmacology Department and Al‐Ameen College of Pharmacy and Rajiv Gandhi University Bangalore India

**Keywords:** diabetic neuropathy, molsidomine, streptozotocin, Wistar rats

## Abstract

Diabetic neuropathy is a disorder that affects various regions of the nervous system and there is no specific treatment available for it. This study evaluated the protective effect of molsidomine in diabetic neuropathy in rats. Diabetes was induced in male Wistar rats by administrating streptozotocin (52 mg/kg ip). Diabetic rats were treated with molsidomine 5 mg/kg po and 10 mg/kg po. After 8 weeks of treatment, motor coordination, mechanical allodynia, mechanical hyperalgesia, nerve conduction velocity, and glycosylated hemoglobin were assessed. Thereafter, animals were killed and the sciatic nerve was isolated for measurement of reduced glutathione and lipid peroxidation, and histopathological analysis. Treatment with molsidomine significantly improved motor coordination, paw withdrawal threshold, mechanical threshold, and nerve conduction velocity. Furthermore, molsidomine treatment also reduced malondialdehyde levels and prevented depletion of reduced glutathione in the sciatic nerve homogenate. Histopathology revealed that molsidomine treatment maintained normal architecture of the sciatic nerve. The results of our study strengthen the alternative use of molsidomine in diabetic neuropathy.

## 1 INTRODUCTION

The prevalence of diabetes mellitus (DM) is increasing at an alarming rate; the number of people with DM was estimated to be 381 million globally in 2013 and 463 million people in 2019 and is projected to increase to 578 million by 2030. It is a disease with high rate of complications such as neuropathy, nephropathy, retinopathy, erectile dysfunction.^1^, ^2^

Diabetic neuropathy is a family of disorders that damage different regions of the nervous system, either individually or in combination. It affects pain fibers, motor neurons, and autonomic nervous system.^3^ It results in large economic costs for its care.^4^, ^5^, ^6^ Various kinds of neuropathy include peripheral neuropathy, proximal neuropathy, autonomic neuropathy, and focal neuropathy.^7^ There are various reasons for the pathogenesis of diabetic neuropathy and the polyol pathway of glucose metabolism is thought as one of the major mechanisms in the pathogenesis of diabetic neuropathy.^8^ Peripheral neuropathy is a type of nerve damage that usually affects feet and legs and sometimes hands and arms.^9^ It is proved that reactive oxygen species (ROS) plays a significant role in the pathophysiology of neuropathic pain in diabetes.^10^ Of all the patients with diabetes , 50% of patients develop neuropathy and painful neuropathy ranges from 10% to 20% in patients with diabetes. Patients with diabetes can experience nerve problems at any time and the problem increases with age, weight, and duration.^5^ The complications across various countries varies from 10% to 30% and it is higher in developed countries than in developing countries. These complications can lead to painful symptoms and can affect the quality of life of patient. The treatment for the painful diabetic neuropathy is mainly focused on pain control. Various treatments, including the use of antidepressants, anticonvulsants, topical agents such as capsaicin cream, lidocaine patches, and isosorbide dinitrate topical spray, have been tried but the benefits were uncertain. Since there is lack of exact medication for neuropathy, research is still ongoing.^2^

Nitric oxide, an endogenous vasodilator acting as a neurotransmitter, is produced from L‐arginine using an enzyme nitric oxide synthase. In patients with diabetes, hyperglycemia stimulates the overproduction of ROS such as superoxide anion which reacts rapidly with nitric oxide radicals forming peroxynitrite anion, a toxic oxidant capable of damaging neurons leading to neuronal injury.^11^, ^12^

Molsidomine is a vasodilating and antianginal agent. As it is a prodrug, it is converted into its metabolite 3‐morpholinosydnonimine (SIN‐1), which spontaneously provides nitric oxide. Nitric oxide has a significant impact on tissue injury, inflammation, vasodilation, cell defense, as well as for regulating cerebrovascular hemodynamics. Nitric oxide also has following characters like antioxidant, antiapoptotic, and anti‐inflammatory activity. It can be used to treat the eye tissue damage caused due to ionizing radiation.^13^, ^14^, ^15^ Until now, there are no data of molsidomine acting against diabetic peripheral neuropathy. Therefore, the present study was designed to evaluate the protective effect of molsidomine against diabetic peripheral neuropathy.

## 2 METHODS

### 2.1 Chemicals

Streptozotocin (STZ; MP Biomedical Pvt. Ltd), ketamine hydrochloride (Neon Laboratories Limited, India), and xylazine (Indian Immunologicals Limited, India) were used in this study. Molsidomine was a gift from Taj Pharmaceutical Pvt. Ltd, India. All other chemicals and reagents used were of analytical grade.

### 2.2 Animals

Wistar rats of appropriate age of either sex weighing about 250‐300g were used in the study. The animals were housed in large propylene cages in an air‐conditioned room at 24 ± 1°C with a 12 hr light/dark cycle and allowed ad libitum access to water and standard diet. Paddy husk was used as bedding material. The use of animals for the experiments were approved by Institutional Animal Ethics Committee (IAEC, reference number: AACP/IAEC/Dec2016/05) and Committee for the Purpose of Control and Supervision of Experimental Animals guidelines were followed.

### 2.3 Induction of diabetes

Streptozotocin was administered at a dose of 52 mg/kg body weight ip to induce diabetes. Glucose solution was given a day after STZ injection. After 72 hours of STZ injection, blood samples (from tail) were collected after overnight fasting. Animals with fasting blood sugar >250m g/dL were considered as diabetic and divided into the following groups:

1. Group 1: Normal Control (n = 6)
2. Group 2: Vehicle‐treated diabetic control (n = 6)
3. Group 3: Diabetic rats treated with molsidomine (5 mg/kg,p.o.) for 8 weeks (n = 6)
4. Group 4: Diabetic rats treated with molsidomine (10 mg/kg, p.o.) for 8 weeks (n = 6).

Non‐diabetic rats were assigned as the normal control group (Group 1).

### 2.4 Motor coordination

Motor coordination was evaluated using a Rota‐Rod treadmill. Rats were initially trained to remain themselves on the rotating rod for more than 2 minutes. In the test session, the rats were placed in the rotating rod and the latency to fall was recorded.^16^

### 2.5 Mechanical allodynia

Mechanical allodynia was performed as per the method described by Yamamoto H et al using a Von Frey apparatus.^17^

### 2.6 Mechanical hyperalgesia

The mechanical nociceptive threshold was measured using a fabricated Randall Selitto paw pressure device, which applies a linearly increasing mechanical forcing to the dorsum of the rat's hind paw.^18^

### 2.7 Nerve conduction velocity

Noninvasive nerve conduction velocity (NCV) was measured using the PowerLab data acquisition system. The rats were anesthetized with ketamine:xylazine(80‐100 mg/kg :5‐10 mg/kg ip, and during the experiment, the body temperature of animal was maintained. The sciatic nerve was stimulated with supramaximal stimuli (8V) at 20 Hz. The latencies of the compound muscle action potentials were recorded via bipolar electrodes from the first interosseous muscle of the hind paw and measured from the stimulus artifact to the onset of the negative M‐wave deflection. Motor NCV was calculated by subtracting the distal latency from the proximal latency and the result were divided by the distance between the stimulating and recording electrodes.

### 2.8 Biochemical studies

#### 2.8.1 Estimation of glycosylated hemoglobin

Blood was withdrawn from retro‐orbital of rat and collected in EDTA tubes. The glycosylated hemoglobin (GHb) was determined using commercially available kits.^19^

### 2.9 Sciatic nerve homogenate preparation

The rats were killed by overdose of anesthesia. A segment of sciatic nerves was isolated. Sciatic nerve samples were rinsed with ice‐cold saline (0.9%w/v sodium chloride) and homogenized in chilled phosphate buffer (pH 7.4). The homogenate thus obtained was used for measurement of reduced glutathione and lipid peroxidation.

### 2.10 Estimation of reduced glutathione

The sciatic nerve was dissected and washed with saline, chopped over ice, and homogenates were prepared with 0.1 mol/L phosphate buffer. Glutathione was quantified using the method described by Moron et al.^20^

### 2.11 Measurement of lipid peroxidation

This parameter was accessed using the method described by Ester bauer and Cheeseman to find out the extent of lipid peroxidation in terms of thiobarbituric acid reactive substances formation. Thiobarbituric acid reacts with malondialdehyde (MDA), a secondary product of lipid peroxidation.^21^

### 2.12 Histopathology

The sciatic nerve was used for histopathological analysis to observe the changes in the cell architecture using hematoxylin and eosin (H&E) stain.

### 2.13 Statistical analysis

All data were expressed as mean ± SEM and were analyzed using one‐way analysis of variance between the groups and followed by Tukey's multiple comparison test to assess differences between the groups. Probability values, *P < .05, **P < .01, and ***P < .001, were considered significant.

## 3 RESULTS

### 3.1 Effect of molsidomine on motor coordination

**FIGURE 1:**
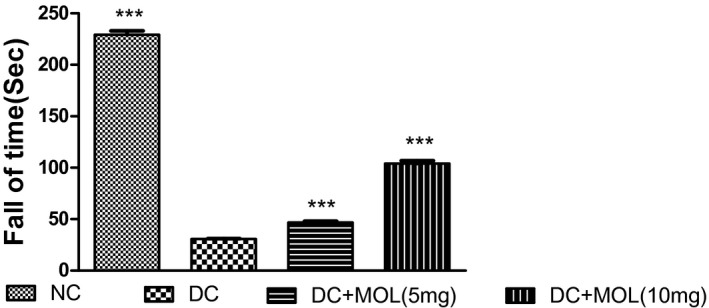
Effect of molsidomine treatment on motor coordination in diabetic rats. Values are represented as mean ± SEM (n = 6). One‐way ANOVA followed by Dunnet's test. ***P < .001 vs diabetic control group. DC, diabetic control; Mol, molsidomine; NC, normal control

The diabetic rats showed a significant decrease in the motor coordination as compared with normal rats (*P* < .001). Pretreatment with molsidomine improved the retention time as compared with diabetic rats. (Figure 1).

### 3.2 Effect of molsidomine on mechanical allodynia

**FIGURE 2:**
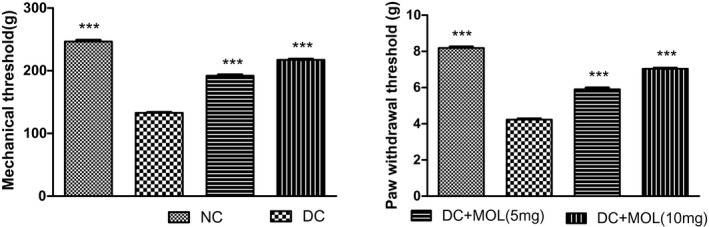
Effect of molsidomine on mechanical threshold and paw withdrawal threshold in diabetic rats. Values are represented as mean ± SEM (n = 6). One‐way ANOVA followed by Dunnet's test. ***P < .001 vs diabetic control group. DC, diabetic control; Mol, molsidomine; NC, normal control

The diabetic rats showed a significant reduction in paw withdrawal threshold as compared with normal rats (*P* < .001). Pretreatment with molsidomine improved paw withdrawal threshold as compared with diabetic rats (Figure 2).

### 3.3 Effect of molsidomine on mechanical hyperalgesia

Diabetic rats showed significant reduction in mechanical threshold as compared with diabetic rats (*P* < .001). Pretreatment with molsidomine improved mechanical threshold as compared with diabetic rats (Figure 2).

### 3.4 Effect of molsidomine on sciatic nerve conduction velocity

**FIGURE 3:**
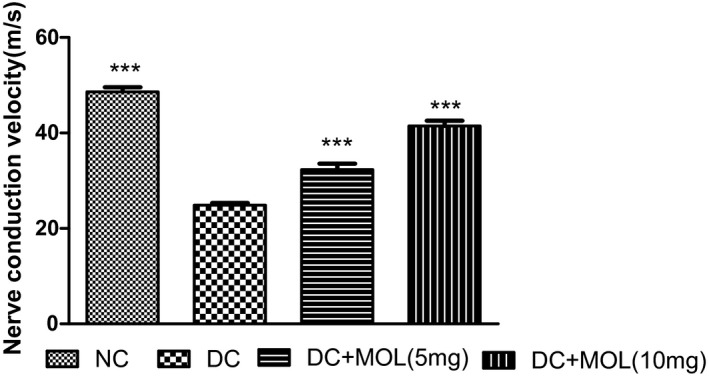
Effect of molsidomine on sciatic nerve conduction velocity in diabetic rats. Values are represented as mean ± SEM (n = 6). One‐way ANOVA followed by Dunnet's test. ***P < .001 vs diabetic control group. DC, diabetic control; Mol, molsidomine; NC, normal control

The NCV was significantly reduced in diabetic control rats when compared with normal rats (*P* < .001). Pretreatment with molsidomine shows significant improvement in NCV when compared with diabetic rats (Figure 3).

### 3.5 Effect of molsidomine on glycosylated hemoglobin, reduced glutathione, and lipid peroxidation

Diabetic rats showed a significant increase in percentage of GHb and MDA levels as compared with normal rats (*P* < .001). Pretreatment with molsidomine significantly reduced percentage of GHb and MDA levels as compared with diabetic control rats (*P* < .001). However, diabetic rats showed a significant reduction of glutathione content in the sciatic nerve when compared with normal rats (*P* < .001). Pretreatment with molsidomine significantly improved glutathione levels as compared with diabetic rats (*P* < .001) (Table 1).

**TABLE 1:** Effect of molsidomine on glycosylated Hb, GSH, and MDA levels in diabetic rats

Sl. No.	Groups	% glycosylated Hb	GSH (mcg/mg)	MDA (nmol/mg)
1.	Normal control	2.76 ± 0.16***	104.2 ± 02.32***	3.28 ± 0.17***
2.	Diabetic control	14.71 ± 0.19	62.89 ± 1.99	7.44 ± 0.55
3.	Diabetic + MOL (5mg/kg)	10.53 ± 0.93***	73.77 ± 0.93***	5.06 ± 0.04***
4.	Diabetic + MOL (10mg/kg)	6.58 ± 0.28***	89.94 ± 0.87***	3.74 ± 0.16***

Note

Values are represented as mean ± SEM (n = 6). One‐way ANOVA followed by Dunnet's test.

Abbreviations: GSH, glutathione; Hb, hemoglobin; mcg, microgram; MDA, malondialdehyde, mg, milligram; MOL, molsidomine; nmol, nanomole.

*** *P* < .001 vs diabetic control group.

### 3.6 Histopathology

**FIGURE 4:**
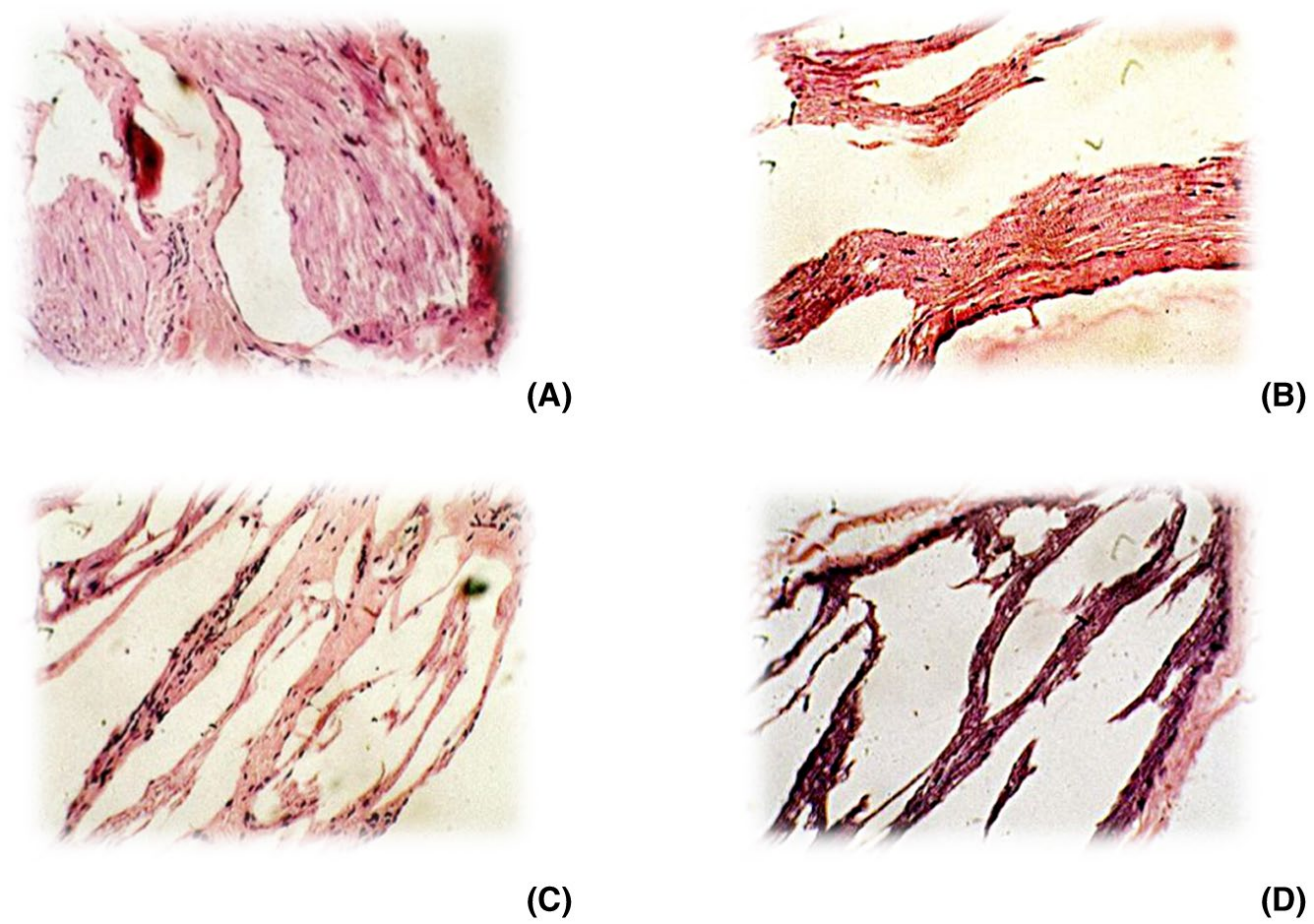
Histopathology of the sciatic nerve (10X). A, Sciatic nerve of normal rats showing elongated Schwann cell nuclei and longitudinally oriented axons with myelin sheath. B, Sciatic nerve of diabetic rats has shown mainly axonal swelling observed with intact myelin. C, Sciatic nerve of molsidomine (5 mg) treated rats showing nuclear degeneration in focal areas with few fiber arrangements. D, Sciatic nerve of molsidomine (10 mg) treated rats showing nuclear degeneration in few areas with few fiber arrangements

The sciatic nerve of normal rats stained with H&E (40X) shown longitudinal section showing the elongated Schwann cell nuclei and longitudinally oriented axons with myelin sheath (Figure 4A). The sciatic nerve of diabetic rats stained with H&E (40X) has shown mainly axonal swelling observed with intact myelin (Figure 4B). The sciatic nerve of rats treated with molsidomine 5 mg/kg showed nuclear degeneration in focal areas with few fiber arrangements (Figure 4C), whereas rats treated with molsidomine 10 mg/kg showed nuclear degeneration in few areas with few fiber arrangements (Figure 4D).

## 4 DISCUSSION

Diabetic neuropathy has to be identified in early stages to prevent secondary complications such as neuropathic pain and diabetic foot.^22^ The most commonly used animal model to study the mechanisms of painful diabetic neuropathy is STZ‐induced diabetic rats. Mechanical allodynia and mechanical hyperalgesia are the common endpoints that are used to assess the analgesic activity of drug in an animal model. In our study, mechanical allodynia and mechanical hyperalgesia were employed to assess the withdrawal threshold of the rat hind paw. The withdrawal threshold was improved following the treatment with molsidomine showing the analgesic activity of the drug.^17^

In STZ‐induced diabetic rats, hyperalgesia is often accompanied by motor incoordination and reduced muscle strength. Therefore, muscle coordination and nerve strength were evaluated by measuring grip strength in STZ‐induced diabetic rats. Diabetic animals showed a significant decrease in grip strength compared with normal rats showing muscle weakness. This study reveals that the treatment with nitric oxide donor molsidomine exerts a positive effect on muscle strength and motor coordination by stimulating soluble guanyl cyclase.^23^, ^24^

Diabetic neuropathy is also accompanied with functional changes in the sciatic nerve and can be evaluated by NCV. In this study, reduction of NCV in the STZ‐treated groups confirmed the abnormal function of the nerve due to diabetes. The molsidomine‐treated groups showed a significant improvement in NCV because sodium ions are pumped from extracellular fluid (ECF) to axoplasm and potassium ions are pumped from axoplasm to ECF and normalization of nerve speed due to decrease in oxidative stress at which an electrochemical impulse propagates down a neural pathway.^25^, ^26^

Excessive generation of ROS and reactive nitrogen species with decreased antioxidant activity leads to development of diabetic neuropathy. Diabetes causes a significant oxidative damage in the sciatic nerve as indicated by an increase in lipid peroxidation and nitric oxide concentration, as well as depletion of antioxidant enzymes.^27^

Previous experimentations have shown that the oxidative stress induced by diabetes causes damage to protein, lipid, DNA, and also causes damages myelin sheath surrounding the nerve. In this study, increase in MDA levels and depletion in reduced glutathione was seen in the sciatic nerve homogenate. The treatment with nitric oxide donor significantly attenuated the oxidative stress markers in the sciatic nerve homogenate of diabetic rats.^28^

Molsidomine after biotransformation by esterases converts into its metabolite SIN‐1, which then liberates nitric oxide and superoxide anion radical. This superoxide anion radical rapids reacts to form peroxynitrite. Literature shows that treatment with molsidomine increases peroxynitrite concentration and decreases superoxide anion radical.^29^

Histopathological analysis of sciatic nerves of diabetic rat showed mainly axonal swelling observed with intact myelin. The treatment with molsidomine significantly improved fiber arrangements and is dose dependent.

## CONFLICT OF INTEREST

There is no conflict of interest.
